# EMS-physicians' self reported airway management training and expertise; a descriptive study from the Central Region of Denmark

**DOI:** 10.1186/1757-7241-19-10

**Published:** 2011-02-08

**Authors:** Leif K Rognås, Troels Martin Hansen

**Affiliations:** 1 The Mobile Emergency Care Unit, Department of Anesthesiology, The Regional Hospital Viborg, Heibergs Allé 4, Postbox 130, 8800 Viborg, Denmark; 2 The Mobile Emergency Care Unit, Department of Anesthesiology, Århus University Hospital, Århus Hospital, Trindsøvej 4-10, 8100 Århus C, Denmark

## Abstract

**Background:**

Prehospital advanced airway management, including prehospital endotracheal intubation is challenging and recent papers have addressed the need for proper training, skill maintenance and quality control for emergency medical service personnel. The aim of this study was to provide data regarding airway management-training and expertise from the regional physician-staffed emergency medical service (EMS).

**Methods:**

The EMS in this part of The Central Region of Denmark is a two tiered system. The second tier comprises physician staffed Mobile Emergency Care Units. The medical directors of the programs supplied system data. A questionnaire addressing airway management experience, training and knowledge was sent to the EMS-physicians.

**Results:**

There are no specific guidelines, standard operating procedures or standardised program for obtaining and maintaining skills regarding prehospital advanced airway management in the schemes covered by this study. 53/67 physicians responded; 98,1% were specialists in anesthesiology, with an average of 17,6 years of experience in anesthesiology, and 7,2 years experience as EMS-physicians. 84,9% reported having attended life support course(s), 64,2% an advanced airway management course. 24,5% fulfilled the curriculum suggested for Danish EMS physicians. 47,2% had encountered a difficult or impossible PHETI, most commonly in a patient in cardiac arrest or a trauma patient. Only 20,8% of the physicians were completely familiar with what back-up devices were available for airway management.

**Conclusions:**

In this, the first Danish study of prehospital advanced airway management, we found a high degree of experience, education and training among the EMS-physicians, but their equipment awareness was limited. Check-outs, guidelines, standard operating procedures and other quality control measures may be needed.

## Background

Prehospital advanced airway management (PHAAM), including prehospital endotracheal intubation (PHETI) continues to be a controversial topic. Some investigators report an alarming rate of complications related to PHAAM, especially to PHETI [1-6], but the results are conflicting, and several other systems reports success rates of PHETI of well over 90% both in American [3] and European [7-16] EMS. Nevertheless: PHAAM is challenging, and recent papers have addressed the need for proper training, skill maintenance and quality control for EMS personnel [11,17-20]. Several guidelines for PHAAM have been published [21-24], stressing the importance of PHAAM-provider experience.

Sollid *et al. *[25] found that there were significant differences between the self-reported experience with difficult PHETI among full-time and part-time HEMS anaesthesiologist working in three different HEMS-schemes in western Norway. Both Sollid [25] and Hüter [26] found room for improvement in HEMS-doctors experience and training in the use of back-up airway devices. Sollid *et al*., by using a predictive Bayesian approach [27,28] to risk management in a HEMS, also found that improving the system and culture regarding PHAAM by introducing risk reducing measures would have a far greater risk reducing potential than focusing on the knowledge and performance of the individual HEMS-physician. Recent work from the Netherlands found that lack of provider coherence to guidelines possesses a potential serious threat to patient safety [29]. An important step in improving quality control in PHAAM is the Utstein style consensus-based template for uniform reporting of data relating to PHAAM, published in 2009 [30]. This template will make it possible to compare PHAAM - data from different EMS's.

The aim of this descriptive study was, as the first from a Danish EMS, to provide baseline PHAAM-data, as suggested by Sollid et al [30]. We focused on EMS-physician training, experience and equipment awareness, as these aspects of PHAAM have been addressed only by a few other papers [31-33], and because knowledge of the present state regarding these aspects may be vital for the improvement of patient safety and for future quality improvement initiatives.

## Methods

### Study population and -area

The eastern and central part of the Central Region of Denmark is an area of approximately 6835 km^2 ^and a population of 835.500 with an overall population density of 122 inhabitants pr. km^2^. It is a mixed urban and rural area, the largest cities being Århus, Randers, Viborg, Silkeborg and Horsens.

### The Emergency Medical System involved

The EMS in this part of the region is a two tiered system. The first tier comprises road ambulances staffed with Emergency Medical Technicians (EMT) on an intermediate or paramedic level (EMT-I/EMT-P). No supraglottic airway devices (SAD) are used by EMTs and they do not perform endotracheal intubation. The second tier comprises Mobile Emergency Care Units (MECU). We studied the MECUs stationed in Århus, Randers, Viborg, Silkeborg and Grenå. The MECUs are rapid response vehicles staffed with a physician and a EMT trained to be the doctors' assistant. The physicians all work in departments of anaesthesia and/or intensive care.

### Inclusion criteria

Doctors working in the physician-staffed EMS in Århus, Silkeborg, Viborg, Randers and Grenå and the medical directors of the same MECU programs.

### Exclusion criteria

Anaesthesiological registrars in Randers who, as part of their training, do limited amount of work in the local EMS.

### Study period and sample size

Questionnaires were sent out in June 2010 to 67 EMS-physicians.

### Variables

The medical directors of the MECU- schemes were contacted in order to obtain information about the actual equipment available, the presence of SOPs, guidelines, checklists and specific training programs regarding PHAAM. A questionnaire (see Additional file 1: *Questionnaire *for a translated version) with both open and closed questions was sent to the physicians. It was an adapted version of the one used by Sollid *et al. *[25].

### Ensuring data quality

The questionnaire was tested for readability and ease of use with the assistance of ten randomly chosen EMS-physicians in Århus (who later received the final version of the questionnaire). To ensure as high a response rate as possible, two reminders were sent by e-mail to the participating physicians.

### Statistics

The material was analysed using descriptive statistics.

### Ethics

The physicians answered the questionnaire anonymously and voluntarily. No patients had their treatment altered because of the study. The protocol has been presented to the regional medical ethics committee, who stated that the study did not need the committee's approval.

## Results

Data from the medical directors showed that the MECUs in this part of the region all have full rapid sequence induction (RSI) -capabilities and carry the same equipment for airway management: Bag -Valve-Mask (BVM) with oxygen reservoir, tracheal tubes and standard laryngoscopes with Miller blades, Airtraq laryngoscope, standard intubating bougie, Gum Elastic Bougies, standard laryngeal masks (LMA), intubating laryngeal masks (ILMA) and equipment for establishing a surgical airway. All airway devices except the Airtraq and the ILMA are available in all sizes from neonatal to large adult. For confirmation of correct laryngeal tube placement all units have capnography available, and all have Weinmann Medumat volume-controlled ventilators.

There are no specific, local protocols, checklists or SOPs and no formal training program for PHAAM. Of the 67 EMS-physicians 53 (79,1%) returned the questionnaire. 52 (98,1%) were specialist in anesthesiology. Their experience and life-support education are shown in Table 1. Of the physicians 45 (84,9%) reported having attended one or more life support course, only 25,5% fulfilled the curriculum suggested by the Danish Society for Anaesthesiology and Intensive Care [34]. 34 (64,2%) had attended one or more course in advanced airway management/management of the difficult airway.

**Table 1:** Self-reported experience and life-support education among EMS-physicians

	Average (range or %)
Years of experience working in anesthesia	17,6 (7 - 33)
Years as a EMS-physician	7,2 (0,3 - 17)
Percentage of total workload spent in EMS	17,5% (5 - 30)
Attended Advanced Trauma Life Support ™(ATLS)	42/53 (79,2)
Attended Advanced Life Support ™(ALS)	26/53 (49,1)
Attended Prehospital Trauma Life Support ™(PHTLS)	18/53 (33,9)
Attended European Pediatric Life Support ™(EPLS)	10/53 (18,9)
None of the above life-support courses	8/53 (15,1)
All of the above life-support courses	5/53 (9,4)
ATLS+ALS +PHTLS (Suggested curriculum by The Danish Society of Anesthesia and intensive Care Medicine) [34]	13/53 (24,5)

The doctors reported a monthly average number of ETI and PHETI of 14,5 and 1 respectively. On average they suggested a minimum of 4,3 ETI/month to maintain the skill.

24 physicians (45,3%) had experienced a difficult PHETI (defined as more than two intubation attempts, a Cormack - Lehan - score of 3 or more, or more than two minutes intubation time) and 20 (37,7%) had been in a situation where PHETI proved impossible. The patient categories in which difficult or impossible PHETI was encountered are summarised in Table 2. Only one (1,9%) of the EMS-physicians had knowledge of any airway management-related deaths within their own EMS.

**Table 2:** Percentage of EMS-physicians who reports having experienced difficult or impossible prehospital endotracheal intubation (PHETI) in different patient categories

		Number (%)
**Difficult PHETI in**	Patient in cardiac arrest	19/53 (35,8)
	Trauma patient	18/53 (33,9)
	Patient with respiratory failure	5/53 (9.4)
	Child	3/53 (5,7)
	Other types of patients	2/53 (3,8)
**Impossible PHETI in**	Patient in cardiac arrest	10/53 (18,9)
	Trauma patient	5/53 (9,4)
	Patient with respiratory failure	1/53 (1,9)
	Child	1/53 (1,9)
	Other types of patients	1/53 (1,9) *

* Patient with epiglotitis

The physicians' awareness of the PHAAM devises available to them is shown in Table 3.

**Table 3:** EMS-physicians knowledge of airway devices available

		Number (%)
**Knows that these devices are available**	Standard Laryngeal Mask	48/53 (90,6)
**(which they are)**	Intubation Laryngeal Mask	45/53 (84,9)
	Gum-Elastic-Bougie	34/53 (64,2)
	Airtraq Laryngoscope	30/53 (56,6)
	Equipment for surgical airway	51/53 (96,2)
	All of the above	15/53 (28,3)
**Thinks that these devices are available**	McCoy laryngoscope	4/53 (7,5 )
**(which they are not)**	Combitube/Larynxtube	2/53 (3,8)
	Set for needle tracheotomy	16/53 (30,2)
**Knows all, and not too many, of the devices available**		11/53 (20,8)

The numbers of EMS-physicians who had received formal training in the use of the different airway devises and the numbers who felt that they had "some" or "considerable" clinical experience in using them, are displayed in Table 4.

**Table 4:** EMS-physicians training and experience with different airway devices

	Have trained Numbers (%)	Have "Some" or "considerable" clinical experience Numbers (%)
**Standard Laryngeal Mask**	51/53 (96,2%)	51/53 (96,2)
**Intubation Laryngeal Mask**	48/53 (90,6%)	39/53 (73,6)
**Gum-Elastic-Bougie**	45/53 (84,9%)	32/53 (60,4)
**Airtraq Laryngoscope**	38/53 (71,7%)	18/53 (34,0)
**Equipment for surgical airway**	52/53 (98,1%)	9/53 (17,0)

The doctors were asked to highlight their preferred airway backup devise in two different clinical scenarios: a "can't intubate - can ventilate situation" and a "can't intubate - can't ventilate situation". The answers are shown in Table 5.

**Table 5:** EMS-physicians' preferred airway backup devices in two different scenarios.

	Can't intubate - can ventilate Numbers (%)	Can't intubate - can't ventilate Numbers (%)
**Bag-mask-valve-ventilation**	14/53 (26,4)	--
**Standard Laryngeal Mask**	9/53 (17,0)	16/53 (30,2)
**Intubation Laryngeal Mask**	35/53 (66,0)	34/53 (64,2)
**Gum-Elastic-Bougie**	25/53 (47,2)	--
**Airtraq Laryngoscope**	15/53 (28,3)	--
**Equipment for surgical airway**	--	30/53 (56,6)
**Other equipment (not available)**	10/53 (18,9)	9/53 (17,0)

## Discussion

This is, to our knowledge, the first study of its kind from a Danish physician-staffed EMS.

The lack of local airway management guidelines or SOPs stands in contrast to what has been reported from for instance London HEMS [15]. It may possess a potential threat to patient safety; it has been shown that SOPs can reduces complications associated with PHAAM and PHETI [11,32]. Whether this applies to practitioners at this level of expertise is to our knowledge not known.

Compared to other physician-staffed EMS/HEMS [7-12,14], the physicians in this study are relatively homogeneous, especially when it comes to speciality; similar to what has been reported from Norway [25] and Göttingen in Germany [13,33].

Few other investigators have reported EMS- physician-experience. We found a higher level of overall experience in anaesthesia than what has been reported from Baden-Württemberg [10], while the HEMS-doctors in Western Norway [25] have more prehospital experience then the EMS- doctors in our region. The EMS-physicians in this study are highly experienced in ETI, performing on average 14,5 ETI/month totally, but only 1 PHETI/month. This total number of ETI/month is considerably more than reported by others [10,25]. Our results, as well as the results of Sollid [25] and Gries [10], demonstrates that prehospital work alone may not be sufficient to maintain adequate PHAAM-/PHETI- skills. This notion is supported by the findings by Fullerton *et al. *[18] showing a higher incidence of airway management problems among HEMS-doctors from specialities where airway management and especially ETI is not part of their day to day work (general practice and surgery) compared to anaesthesiologists and emergency physicians.

In our study, the physician - reported incidence of difficult or impossible PHETI ("non-intubation situation") is low and deaths related to PHAAM apparently very rare compared to the findings of Sollid [25]. We believe that this is mainly due to the doctors' extensive experience. This is supported by the findings of Combes *et al. *[11], demonstrating a higher incidence of PHAAM-problems among non-specialist working in the EMS as opposed to consultants. The recently published guidelines [21-24], as well as the 2008 Cochrane review [19] also emphasises the importance of a high degree of operator experience and skill-maintenance in PHAAM and PHETI.

The equipment available for the physicians in this study is more extensive than what has been reported by others, as highlighted in Table 6. We have found no other study addressing the question of EMS- physician equipment awareness. Knowing one's options when it comes to PHAAM seems vital, and it may be especially critical for the physicians who (wrongly) think that for instance the McCoy Laryngoscope is available and plans his/her actions accordingly. The relatively poor equipment awareness in this study may be explained by the lack of formal introductory programs, both for new physicians and when new equipment is introduced. Mandatory teaching and check-out procedures may be needed as the lack of equipment awareness may pose a threat to patient safety.

**Table 6:** The availability of different airway back-up device as reported by other investigators

	Laryngeal Mask	Intubation Laryngeal Mask	Larynxtube	Combitube	Gum-elastic-bougie	Surgical airway
Hüter (Thuringia,D) [29]	36		10	5		100
Schmid (Baveria, D) [24]	26	7	26	18		71
Genzwürker (Baden- Württemberg, D) [20]	51	1	68,3*			70
Timmermann (Northern Germany) [26]	37,1	6,1	15,5*			57,8
Schmid (UK) [30]	73	8	23*		69	62
Current study (DK)	100	100	0	0	100	100

Numbers are percentage of the investigated EMS/HEMS in each study who carry the device

* Larynxtube and Combitube reported together

The physicians training with the airway devices is in general satisfactory and in line with what has been reported from anaesthesiologists working as EMS-physicians in northern Germany [33]. The level of expertise is considerably higher than that reported for non- anaesthesiological EMS-physicians [33]. The reported clinical experience in the use of especially the LMA and the ILMA, but also the Gum-Elastic-Bougie, is considerable, and our results correspond well with those of the part-time employed HEMS - doctors in western Norway [25]. This part of our study further supports the notion that when it comes to anaesthesiologist achieving and maintaining experience in advanced airway management, it may be better to be employed both in- and pre-hospital, rather than working full-time in the EMS/HEMS.

Most of the EMS - physicians rely on their clinical work for maintaining airway management skills and 75,5% know that this is left to their own discretion as is the case for their Norwegian [25] and some German [26] colleagues. This differs from what has been reported from the UK [15,16].

Again, a uniform training and certification system for all EMS-physicians may be necessary to ensure a minimum of ongoing training and clinical experience with the available equipment [35].

We found that the ILMA, followed by the surgical airway, is the most favoured back-up devices in a "can't intubate - can't ventilate situation". To our knowledge, this kind of data has not been reported before. Our findings are not in complete accordance with the guidelines for treatment of the unexpected difficult airway [36], which recommends the use of a standard LMA or a surgical airway in these situations. In the "can't intubate - can ventilate situation" following RSI, the guidelines [36] recommend oxygenation using BVM-ventilation or a standard LMA and awakening the patient while postponing surgery if possible. These possible deviations from the guidelines may be due to the fact that awakening the patient is often not a very attractive option in the prehospital setting. They, as well as the considerable variation among the physicians' preferred back- up devices, may however also be due to the lack of SOPs, guidelines and standardised PHAAM-training in the investigated EMS. Again this seems to be a point of possible improvement in the programs in this study.

A limitation, but also a strength of this study is that the data comes from the EMS-physicians themselves. The true frequency of PHAAM/PHETI in our schemes is not known, nor is the rate of complications. Our results reflect the physicians' perception of their work. Recall bias and a (subconscious) denial of one's own shortcomings cannot be ruled out. Gathering more precise and prospective data related to PHAAM should be a priority in the following years.

The response rate in this study is satisfactory, and we have no reason to believe that the characteristics of the repliers should be different from those of the whole group of EMS physicians. Nevertheless, selection bias cannot be ruled out.

We primarily used fixed response questions, thus minimizing the risk of instrument bias.

Most of the MECUs in Denmark operate with case-loads, staffing, staff-education and call-out-criteria that are comparable to those of the programs investigated in this study. And even though the number of EMS-physicians in this study is limited, we believe that our results are representative for most Danish MECUs. We also believe that the challenges of low PHAAM equipment awareness, lack of formal PHAAM training, lack of local guidelines and SOPs identified in this study may be applicable to EMS/HEMS in other countries as well, especially those with a similar organisation to the one in this study, e.g. EMS/HEMS in Norway, Finland, Germany, The Netherlands, Switzerland, Austria and France.

## Conclusion

In this first Danish study of prehospital advanced airway management, we found that the anaesthesiologists working as part-time EMS- physicians in the central and eastern part of The Central Region of Denmark are highly experienced in endotracheal intubation.

They have a high degree of education and training in the use of back-up devices for airway management, but their equipment awareness is limited. The EMS in this study did not have formal training programs regarding PHAAM, nor did they have any local airway management guidelines, checklists or SOPs. Improvement on an organisational level may be needed to ensure patient safety.

Prospective studies, using the new Utstein template [30] for collecting a standardised set of data, are wanted; both to establish baseline of prehospital advanced airway management in different EMS and to measure the effect of interventions, such as the implementations of check-outs, guidelines, SOPs and other quality control measures.

## Competing interests

The authors declare that they have no competing interests.

## Author information

**LKR **is a consultant anaesthesiologist and an EMS-physician in Viborg and Århus, DK. He is Program Director of the Scandinavian Society of Anaesthesiology and Intensive Care Medicine (SSAI) Program in Critical Emergency Medicine.

**TMH **is a consultant anaesthesiologist and medical director (on leave) of the Mobile Emergency Care Unit in Århus, DK. He is currently working as a HEMS-physician in the East Anglian Air Ambulance, UK.

## Authors' contributions

**LKR **conceived the study and designed the questionnaire, managed and analyzed the data and drafted the manuscript.

**TMH **helped conceive the study and participated in the design of both the study and the questionnaire.

Both authors have read and approved the final manuscript.

## Supplementary Material

Additional file 1 **A translated version of the questionnaire used to gather the data from the EMS-physicians in this study is provided as Additional file 1*: Questionnaire***. Click here for file

## References

[B1] Katz SH Falk JL Misplaced endotracheal tubes by paramedics in an urban emergency medical services system Ann Emerg Med 2001 37 32 37 10.1067/mem.2001.112098 11145768

[B2] Wirtz D Ortiz C Newman D Zhitomirsky I Unrecognized misplacement of endotracheal tubes by ground prehospital providers Prehosp Emerg Care 2007 11 213 218 10.1080/10903120701205935 17454811

[B3] Germann CA Baumann MR Kendall KM Performance of Endotracheal Intubation and Rescue Techniques by Emergency Services Personnel in an Air Medical Service Prehospital Emergency Care 2009 13 1 44-49 10.1080/10903120802474505 19145523

[B4] Wang H Cook LJ Chang CC Yealy D Lave J Outcomes after out of-hospital endotracheal intubation errors Resuscitation 2009 80 50 55 10.1016/j.resuscitation.2008.08.016 18952357

[B5] Denver Metro Airway Study Group A prospective multicenter evaluation of prehospital airway management performance in a large metropolitan region Prehosp Emerg Care 2009 13 304 31 10.1080/10903120902935280 19499465

[B6] Cobas M De la Peña M Manning R Candiotti C Varon AJ Prehospital Intubations and Mortality: A Level 1 Trauma Center Perspective Anesth Analg 2009 109 489 93 10.1213/ane.0b013e3181aa3063 19608824

[B7] Adnet F Jouriles NJ Le Toumelin P Hennequin B Tailandier C Rayeh F Couvreur J Nougiere B Nadiras P Ladka A Fleury M Survey of out-of-hospital emergency intubations in the French prehospital medical system: a multicenter study Ann Emerg Med 1998 32 454 60 10.1016/s0196-0644(98)70175-1 9774930

[B8] Thierbach A Piepho T Wolcke B Küster S Dick W [Prehospital emergency airway management procedures. Success rates and complications.] Anaesthesist 2004 53 543 550 10.1007/s00101-004-0679-z 15088093

[B9] Slagt C Zondervan A Patka P de Lange JJ A retrospective analysis of the intubations performed during 5 years of helicopter emergency medical service in Amsterdam Air Med J 2004 23 36 37 10.1016/j.amj.2004.06.004 15337954

[B10] Gries A Zink W Bernhard M Messelken M Schlechtriemen T Realistic assessment of the physician-staffed emergency services in Germany Anaesthesist 2006 55 1080 1086 10.1007/s00101-006-1051-2 16791544

[B11] Combes X Jabre P Jbeili C Leroux B Bastuji-GArin S Margenet A Adenet F Dhonneur G Prehospital Standardization of Medical Airway Management: Incidence and Risk Factors of Difficult Airway Acad Emerg Med 2006 13 8 10.1197/j.aem.2006.02.016 16807397

[B12] Schmid MC Deisenberg M Strauss H Schûttler J Birkholz T [Equipment of a land-based emergency medical service in Bavaria. A questionnaire.] Anaesthesist 2006 55 1051 1057 10.1007/s00101-006-1078-4 16906427

[B13] Timmermann A Eich C Russo SG Natge U Bräer A Rosenblatt WH Braun U Prehospital airway management: a prospective evaluation of anaesthesia trained emergency physicians Resuscitation 2006 70 179 85 10.1016/j.resuscitation.2006.01.010 16828956

[B14] Timmermann A Russo SG Eich C Roessler M Braun U Rosenblatt WH Quintel M The Out-of-Hospital Esophageal and Endobronchial Intubations Performed by Emergency Physicians Anesth Analg 2007 104 619 23 10.1213/01.ane.0000253523.80050.e9 17312220

[B15] Newton A Ratchford A Kahn I Incidence of adverse events during prehospital rapid sequence intubation: A review of one year on the London Helicopter Emergency Medical Service J Trauma 2008 64 487 492 10.1097/TA.0b013e31802e7476 18301219

[B16] Schmid M Mang H Ey K Schütler J Prehospital airway management on rescue helicopters in the United Kingdom Anaesthesia 2009 64 625 631 10.1111/j.1365-2044.2008.05859.x 19453316

[B17] Paal P Herff H Mitterlechner T von Goedcke A Brugger H Lindner KH Wenzel V Anaesthesia in prehospital emergencies and in the emergency room Resuscitation 2010 81 148 154 10.1016/j.resuscitation.2009.10.023 19942337

[B18] Fullerton JN Roberts KJ Wyse M Should non-anaesthetists perform pre-hospital rapid sequence induction? an observational study Emerg Med J Published Online First 26 July 2010 10.1136/emj.2009.086645 20660897

[B19] Lecky F Bryden D Little R Tong N Moulton C Emergency intubation for acutely ill and injured patients Cochrane Database Syst Rev 2008 CD001429 10.1002/14651858.CD001429.pub2 PMC7045728 18425873

[B20] Herff H Wenzel V Lockey D Prehospital intubation: the right tools in the right hands at the right time Anesth Analg 2009 109 303 305 10.1213/ane.0b013e3181ad8a1e 19608796

[B21] Davies DP Prehospital intubation of brain-injured patients Curr Opin Crit Care 2008 14 142 8 10.1097/MCC.0b013e3282f63c40 18388675

[B22] Wang HE O'Connor RE Domeier RM National Association of EMS Physicians Position paper-- Prehospital Rapid Sequence Intubation Prehosp Emerg Care 2001 5 40 48 10.1080/10903120190940317 11194068

[B23] Kupas DF Greenwood MJ Pinchalk ME Mullins T Gluckman W Sweeney TA Hostler D An Algorithmic Approach to Prehospital Airway Management Prehosp Emerg Care 2008 9 145 155 10.1080/10903120590924618 16036838

[B24] Berlac P Hyldmo PK Kongstad P Kurola J Rostrup Nakstad A Sandberg M Prehospital airway management - guidelines from a task force from the Scandinavian society for anaesthesiology and Intensive care Medicine Acta Anaesthesiol Scand 2008 52 897 907 10.1111/j.1399-6576.2008.01673.x 18702752

[B25] Sollid SJM Heltne JK Søreide E Lossius HM Pre-hospital advanced airway management by anaesthesiologists: Is there still room for improvement? Scand J Trauma, Resuscitation, Emerg Med 2010 2008 15 2 10.1186/1757-7241-16-2 PMC2556637 18957064

[B26] Hüter L Schreiber T Reichel J Schwarzkopf K Present-day prehospital airway management in the former Eastern German state of Thuringia: equipment and education of emergency physicians European J Emerg Med 2009 16 97 99 10.1097/MEJ.0b013e32830a7577 19177026

[B27] Sollid SJM Lossius HM Nakstad AR Aven T Søreide E Risk assessment of pre-hospital trauma airway management by anaesthesiologists using the predictive Bayesian approach Scand J Trauma, Resuscitation, Emerg Med 2010 18 22 10.1186/1757-7241-18-22 PMC2873366 20409306

[B28] Sollid SJM Lossius HM Søreide E Pre-hospital intubation by anaesthesiologists in patients with severe trauma: an audit of a Norwegian helicopter emergency medical service Scand J Trauma, Resuscitation, Emerg Med 2010 18 30 10.1186/1757-7241-18-30 PMC2903496 20546578

[B29] Franschman G Peerdeman SM Greuters S Vieveen J Brinkman AC Christiaans HM Toor EJ Jukema GN Loer SA Boer C Prehospital endotracheal intubation in patients with severe traumatic brain injury: guidelines versus reality Resuscitation 2009 80 1147 1151 10.1016/j.resuscitation.2009.06.029 19632024

[B30] Sollid, Lockey, Lossius and Prehospital advanced airway management expert group A consensus-based template for uniform reporting of data from pre-hospital advanced airway management Scand J Trauma, Resuscitation, Emerg Med 2009 17 58 10.1186/1757-7241-17-58 PMC2785748 19925688

[B31] Genzwürker H Lessing P Ellinger K Viergutz T Hinkelbain J [Infrastructure of emergency medical services. Comparison of physician-staffed ambulance equipment in the state of Baden-Wuerttemberg in 2001 and 2005.] Anaesthesist 2007 56 665 672 10.1007/s00101-007-1194-9 17483912

[B32] Helm M Hossfeld B Schäfer S Hoitz J Lampl L Factors influencing emergency intubation in the pre-hospital setting--a multicentre study in the German Helicopter Emergency Medical Service Brit J of Anaesth 2006 96 67 71 10.1093/bja/aei275 16311285

[B33] Timmermann A Braun U Panzer W Schaeger M Schnitzker M Graf BM [Out-of-hospital airway management in northern Germany. Physician-specific knowledge, procedures and equipment.] Anaesthesist 2007 56 328 334 10.1007/s00101-007-1153-5 17334740

[B34] The Scandinavian Society of Anaesthesiology and Intensive Care Medicine http://www.dadlnet.dk/master/kunder/dokument/m742/u723/praehospital-APRIL-09.pdf

[B35] Konrad C Schupfer G Wietlisbach M Gerber H Learning manual skills in anesthesiology: is there a recommended number of cases for anesthestic procedures ? Anesth Analg 1998 86 635 639 10.1097/00000539-199803000-00037 9495429

[B36] Henderson JJ Popat MT Latto IP Pearce AC Difficult Airway Society guidelines for management of the unanticipated difficult intubation Anaesthesia 2004 59 675 694 10.1111/j.1365-2044.2004.03831.x 15200543

