# Supplementary material for: EMS-physicians' self reported airway management training and expertise; a descriptive study from the Central Region of Denmark
Source: Scand J Trauma Resusc Emerg Med. 2011 Feb 8;19:10. doi: 10.1186/1757-7241-19-10 (PMC3045910; doi:10.1186/1757-7241-19-10)
Supplement: Additional file 1 — A translated version of the questionnaire used to gather the data from the EMS-physicians in this study is provided as Additional file 1: Questionnaire. [file 1757-7241-19-10-S1.PDF]

Prehospital advanced airway management in the Central Region of Denmark

Leif Rognås, Troels Martin Hansen

1.	Are you a specialist in anaesthesiology?	☐ No<br>☐ Yes
2.	How many years of experience in anaesthesiology do you have?	____________ years
3.	How many years of prehospital experience do you have?	____________years
4.	How many percent of your work do you spend prehospital ?	____________%
5.	In which of these courses have you taken part?	☐ PHTLS<br>☐ ATLS<br>☐ EPLS<br>☐ ALS<br>☐ SSAI Course in advanced airway management<br>☐ Airway management course during residency<br>☐ Other ________________________
6.	Have you taken part in any airway management course during the last four years??<br><br>If yes, what course / courses?	☐ No<br>☐ Yes<br><br>______________________________<br>______________________________
7.	How many endotracheal intubations do you perform on average pr. month?	In-hospital:____________ Prehospital:______________
8.	Do you feel that there is a minimum requirement for endotracheal intubations pr. month necessary to maintain this skill?<br><br>**If yeas – how many?**<br>**If no – why not?**	☐ No<br>☐ Yes<br>______________________________<br>______________________________<br>______________________________<br>_____________________________
9.	Have you ever experienced a difficult prehospital endotracheal intubation?<br>(Definition: >2 intubation attempts **or** >2 minutes used **or** Cormac – Lehan Score ≥3 **or** intubation of the oesophagus)<br><br>**If yes – in what type of patient / patients?**	☐ No<br>☐ Yes<br>☐ Trauma<br>☐ Cardiac arrest<br>☐ Respiratory failure<br>☐ Child<br>☐ Other________________________________

10. Have you ever had to give up on a prehospital endotracheal intubation?<br><br>**If yes – how did you solve the problem?**<br><br>**If yes – in what type of patient / patients?**	☐ No<br>☐ Yes – problem solved by bag-valve-mask ventilation<br>☐ Yes – problem solved with a supraglottic device<br>☐ Yes – problem solved with a transtracheal airway<br>☐ Yes – problem solved in another way<br>☐ Yes – and the problem could not be solved prehospital<br><br>☐ Trauma<br>☐ Cardiac arrest<br>☐ Respiratory failure<br>☐ Child<br>☐ Other________________________________
11. Are you aware of any prehospital deaths related to airway management in your prehospital program?	☐ No<br>☐ Yes
12. What type of airway management equipment is available in your prehospital program?	**Supraglottic devices**<br>☐ Laryngeal masks (LMA)<br>☐ LMA Proseal<br>☐ I-Gel<br>☐ Intubating laryngeal mask (ILMA / Fasttrach)<br>☐ Larynxtube<br>☐ Combitube<br>☐ Other ____________________<br><br>**Intubation aids**<br>☐ McCoy laryngoscope<br>☐ Gum-Elastic-Bougie<br>☐ AirTraq<br>☐ Other____________________<br><br>**Transtracheal devices**<br>☐ Equipment for transtracheal needle oxygenation<br>☐ Equipment for establishing a surgical airway<br>☐ Other____________________<br><br>☐ Don't know
13. Which of these airway devices have you received any form of education or training in using?	**Supraglottic devices**<br>☐ Laryngeal masks (LMA)<br>☐ LMA Proseal<br>☐ I-Gel<br>☐ Intubating laryngeal mask (ILMA / Fasttrach)<br>☐ Larynxtube<br>☐ Combitube<br>☐ Other ____________________<br><br>**Intubation aids**<br>☐ McCoy laryngoscope<br>☐ Gum-Elastic-Bougie<br>☐ AirTraq<br>☐ Other____________________<br><br>**Transtracheal devices**<br>☐ Equipment for transtracheal needle oxygenation<br>☐ Equipment for establishing a surgical airway<br>☐ Other____________________<br>☐ None of the above

14. What's your clinical experience in the use of the following airway devices?

Laryngeal mask (LMA)
☐ None
☐ Minor clinical experience
☐ Some clinical experience
☐ Considerable clinical experience

LMA Proseal
☐ None
☐ Minor clinical experience
☐ Some clinical experience
☐ Considerable clinical experience

I-Gel
☐ None
☐ Minor clinical experience
☐ Some clinical experience
☐ Considerable clinical experience

Intubating Laryngeal mask (ILMA / Fasttrach)
☐ None
☐ Minor clinical experience
☐ Some clinical experience
☐ Considerable clinical experience

Larynxtube
☐ None
☐ Minor clinical experience
☐ Some clinical experience
☐ Considerable clinical experience

Combitube
☐ None
☐ Minor clinical experience
☐ Some clinical experience
☐ Considerable clinical experience

McCoy laryngoscope
☐ None
☐ Minor clinical experience
☐ Some clinical experience
☐ Considerable clinical experience

Gum – Elastic - Bougie
☐ None
☐ Minor clinical experience
☐ Some clinical experience
☐ Considerable clinical experience

AirTraq
☐ None
☐ Minor clinical experience
☐ Some clinical experience
☐ Considerable clinical experience

Equipment for transtracheal needle oxygenation
☐ None
☐ Minor clinical experience
☐ Some clinical experience
☐ Considerable clinical experience

Equipment for establishing a surgical airway
☐ None
☐ Minor clinical experience
☐ Some clinical experience
☐ Considerable clinical experience

Other
☐ None
☐ Minor clinical experience
☐ Some clinical experience
☐ Considerable clinical experience

15. What is your preferred airway management device in a **"Can ventilate – can't intubate"**-situation?

Supraglottic devices
☐ Bag-valve-mask
☐ Laryngeal masks (LMA)
☐ LMA Proseal
☐ I-Gel
☐ Intubating laryngeal mask (ILMA / Fasttrach)
☐ Larynxtube
☐ Combitube
☐ Other ____________________

Intubation aids
☐ McCoy laryngoscope
☐ Gum-Elastic-Bougie
☐ AirTraq
☐ Other____________________

Transtracheal devices
☐ Equipment for transtracheal needle oxygenation
☐ Equipment for establishing a surgical airway
☐ Other____________________

16. What is your preferred airway management device in a **"Can't ventilate – can't intubate"**-situation?

Supraglottic devices
☐ Laryngeal masks (LMA)
☐ LMA Proseal
☐ I-Gel
☐ Intubating laryngeal mask (ILMA / Fasttrach)
☐ Larynxtube
☐ Combitube
☐ Other ____________________

Intubation aids
☐ McCoy laryngoscope
☐ Gum-Elastic-Bougie
☐ AirTraq
☐ Other____________________

Transtracheal devices
☐ Equipment for transtracheal needle oxygenation
☐ Equipment for establishing a surgical airway
☐ Other___________________

17. How do you maintain your airway management skills?

☐ Regularly clinical work
☐ Mannequin training
☐ Training on cadavers
☐ Simulations
☐ Airway management courses
☐ Other ____________________
☐ None of the above

18. Does your prehospital program carry out prehospital airway management education and -training on a regular basis?

☐ No
☐ Yes
☐ Don't know

19. Does your prehospital program have written guidelines for prehospital airway management?

☐ No
☐ Yes, national / international guidelines are implemented
☐ Yes there are local, specific guidelines for prehospital airway management
☐ Don't know
